# Financial burden and quality of life of informal caregivers of patients with wet age-related macular degeneration

**DOI:** 10.1186/s13561-016-0116-4

**Published:** 2016-08-26

**Authors:** Hannah Weyer-Wendl, Peter Walter

**Affiliations:** Department of Ophthalmology, RWTH Aachen University, Pauwelsstr. 30, 52074 Aachen, Germany

**Keywords:** Age-related macular degeneration, Caregivers, Costs, Quality of life

## Abstract

**Purpose:**

The purpose of this research is to quantify the cost burden, care times and the impact on the quality of life (QoL) of informal caring relatives caring for patients with wet age-related macular degeneration (wet AMD). Moreover we investigated the impact of care times on the QoL.

**Methods:**

Through a specifically designed questionnaire, 150 caring relatives were interviewed retrospectively on all accrued financial costs, caring times incurred and the current QoL, assessed by a Visual Analogue Scale for happiness (VAS).

**Results:**

The caring time incurred was on average 6.4 ± 8.5 (mean +/- SD) hours per week. The QoL was on average rated at 6.7 ± 1.9 on a ten point scale. Financial strain was incurred by the direct non-medical costs of on average € 405 ± 1104 and the direct medical costs of on average € 134 ± 340 per year. Indirect costs were stated by two caregivers as amounting to € 2400 and € 6000 net income loss per year respectively. Caregivers of privately insured patients with wet AMD carried a financial cost burden which was up to six times higher than caregivers of patients who were on state insurance while showing the same visual acuity.

**Conclusion:**

The evaluation shows that caregivers of privately insured patients with wet AMD have higher costs than caregivers of patients with state insurance coverage. This burden seems to be a factor to be considered independently since it does not appear to have any relation to patients AMD acuity.

## Background

It is well known that by 80 years old, more than 80 % of the main causes of blindness are due to age-related conditions such as age-related macular degeneration (AMD), cataract and glaucoma [1]. Due to our increasing elderly population, resource allocation for the treatment and monitoring of these potentially blinding conditions has been expanded. Epidemiological studies in AMD were able to provide information on the prevalence of the condition and demonstrate a six-fold risk of developing AMD at the age of 70–79, growing to 25-fold in persons over 80 in comparison to the 55–69 age group [2]. The treatment results have changed from stabilising vision using laser photocoagulation or photodynamic therapy to improving vision using intravitreal injections with anti-vascular endothelial growth factor (anti-VEGF) [3]. AMD represents a major public health priority for global health systems, but there are only very few studies, such as the study of Bandello et al., Javitt et al. or Pauleikoff et al., that assess the economic burden.

AMD has a profound effect on patients’ QoL, especially once the disease has progressed to its late stages as is the case with wet AMD [4–7]. As vision loss causes a higher need for care, an important aspect worth exploring is how much the quality of life of caregivers is affected. The fact that patients with degrading vision can, to a certain extent, overcome functional difficulties – for example by using magnifying glasses, screen-projection devices or other low-vision aids and devices – makes it interesting to figure out if the caregiver cost burden correlates with the vision loss of the affected patient. The burden on patients themselves has already been subject to plenty of research. They have shown that medical and non-medical costs of patients with wet AMD are incurred on a considerable scale [8, 9]. Regarding the caregiving relatives, it is known that the visual impairment of patients contributes to a 35.4 % higher risk of depression in said relatives. Furthermore, the ability to deal with social problems and the overall life satisfaction is reduced in relatives tasked with caring for the patients [10]. The increased costs of patients affected by wet AMD stands in direct correlation with their remaining visual acuity [11, 12]. Although it is acknowledged that the role of informal caregivers is important, little is known about the consequences of giving care to patients with wet AMD. Considering these findings and against the background of the increasing costs for the medical treatment, knowing to what extent caregivers are financially burdened and which strains are placed upon their QoL is very valuable. The primary target of the study was to quantify and analyse the burden placed on the caregiver regarding incurred financial resources, invested time and the QoL. Subsequently, tests were carried out to determine whether the caregiver burden was associated with

- the visual acuity of the patient,
- the caregiver gender,
- the intensity of caregiving.

## Methods

For this study, 150 caring relatives of 150 patients with wet AMD were recruited over the course of 18 months, from 1 January 2013 to 31 July 2014, at the Department of Ophthalmology, RWTH Aachen University. The interviews were all conducted at the Department by the same research assistant under GCP-compliant conditions, since the patients and the caregivers were informed by an information sheet and have signed the declaration of consent. Almost every patient and caregiver was interested in participating in this study. The study was approved by the Ethics Committee of the University Hospital Aachen. The patients were recruited regardless of the patient’s age and visual acuity. Patient inclusion criteria were a confirmed diagnosis of wet AMD and permanent residence in the Federal Republic of Germany. Patients were excluded if they had other diseases requiring care by professionals or caregivers. Inclusion criteria for the caregivers were an existing family relationship with the patient as well as the ability to communicate in German and a minimum age of 18 years. In this study, all potential costs arising on the relatives’ side were structured using health economic classification methods subdivided into three groups of costs; direct costs, indirect and intangible costs. The caregivers were asked once about their costs during different time periods (last 3 weeks, last 3 month, last year and last 3 years), which were calculated to represent the caregiver cost of 1 year.

### Questionnaire

The questionnaire used in this study is composed of 16 pages and was completed independently by the caregivers in approximately 30 min.

First of all, questions were asked about the intensity of the care given to the patients. Here, the average hours of care per week and care characteristics were noted. Then inquiries about the caregivers’ QoL were made using the Visual Analogue Scale (VAS). The VAS had already proveen to be easily applicable and understandable. It facilitates surveying the impact on caregivers’ QoL [13]. Using the VAS, the caregivers could indicate their QoL on a scale from 0 (totally unhappy) to 10 (perfectly happy). In addition to the VAS, the QoL was measured using the Caregiver Reaction Assessment-Questionnaire (CRA). The 24 questions of the CRA measured the caregivers self-esteem (7 questions), impact on schedule (5 questions), lack of family support (5 questions), impact on finances (3 questions) and impact on health (4 questions) [14]. A descriptive analysis and a principal component analysis of the CRA can be viewed in another publication by the same authors [15]. In the questionnaire, the CRA was followed by data on a wide variety of doctors’ visits – for which the patients had to be accompanied by the caregivers; the data included the number of visits, the distances covered and the time spent. Further costs – such as travel costs, parking fees, costs for medication and visual aids, individual health services and co-payments – were also captured, providing that these costs were not reimbursed and were paid for by the caregiver, since the cost calculation was made from the perspective of the caregiver. Furthermore, the time spent on daily activities by caregivers (household chores, leisure activities and office work) were identified and quantified. Finally, socio-demographic data of the caregivers – such as age, family relationship to the patient, employment status, household income, living situation and marital status – were recorded. Patient data – such as visual acuity, diagnosis, previous therapies and visual co-morbidities – were also transferred from the patient records to the questionnaire. The resource consumption (e.g. time caring hours per week) was transferred to the calculated costs. For this purpose in particular the coefficient of determination was used to indicate the proportion of the variance in the dependent variable that is predictable from the independent variable. The intangible costs were calculated through the given values on the VAS.

### Statistical evaluation

All analyses were conducted using SAS 9.2 (SAS Institute Inc., Cary, North Carolina) with a significance level of 5 %. Categorical data are presented by frequencies and percentages. Continuous variables are presented as mean values ± standard deviation (SD). For the statistical analysis of the data, the Kruskal-Wallis-test, Wilcoxon-test, *t*-test, linear regression, chi-square test and multivariate ANCOVA were used as appropriate.

## Results

### Participants

150 caregivers of 150 patients with wet AMD were included in the study. The missing values amounted to 3.3 %. The caregivers consisted of a group of 92 women (61 %) and 58 men (39 %). The average age was 60 years and was distributed between 18 and 83 years. 49 % of caregivers were younger than 60 years. Most caregivers were spouses (47 %) or children (39 %) of the patients. The patients consisted of a group of 79 women (53 %) and 71 men (47 %). The patient age was, on average, 77 years. The patients’ visual acuity of the better eye was divided into three groups: (1) 58.6 % had a visual acuity of >0.3, (2) 18 % had a visual acuity between ≤ 0.3 and >0.1, and (3) 35.3 % had a visual acuity of ≤ 0.1. The division into these groups was made after consultation with the ophthalmologists of the eye clinic. Most patients (81 %) were insured by the state; the remaining patients (19 %) were privately insured. An overview of the socio-demographic data can be found in Table 1.

**Table 1:** Socio-demographic data of patients and caregivers

Variable	*n*	(%)
Patients		
Age (years) (Mean 77.17) (SD 7.72)		
Male	71	47
Female	79	53
Diagnosis in addition to wet AMD		
Glaucoma	12	8
Cataract	54	36
Diabetes	9	6
Caregivers		
Age (years) (Mean 60.66) (SD 15.24)		
Male	58	39
Female	92	61
Work situation		
Retired	74	49
Employed	55	37
Housewife/man	12	8
Unemployed	9	6
Relationship to the patient		
Spouse	70	47
Son/Daughter	59	39
Son-/Daughter-in-law	9	6
Grandchild	6	4
Others	6	4

### Caring time

**Fig. 1:**
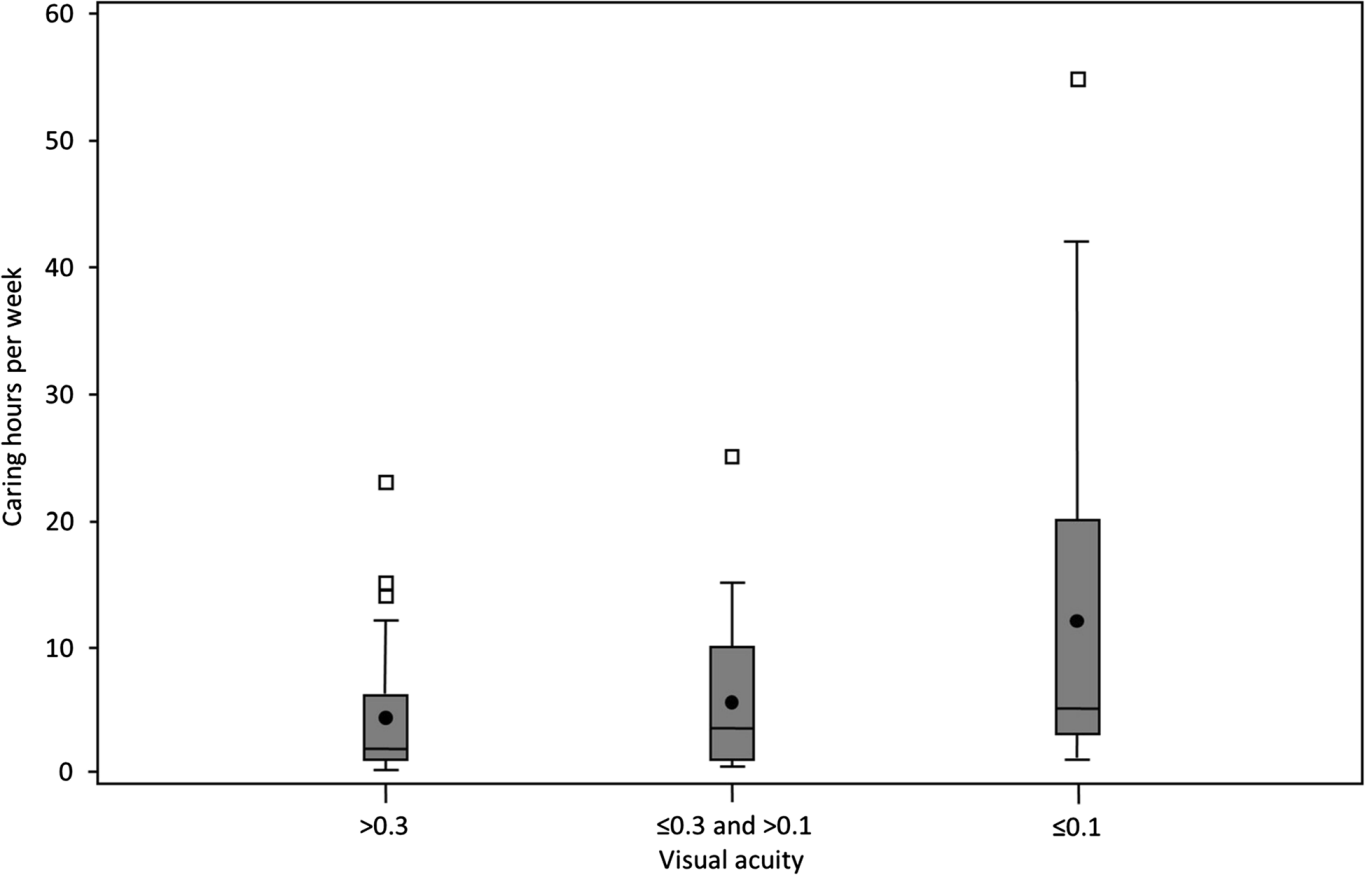
Three boxplots show the significant interdependence between the absolute number of caring hours per week and the visual acuity (Kruskal-Wallis *p*-value <0.01). A visual acuity of >0.3 caused on average 4.3 ± 4.4, a visual acuity of ≤0.3 - >0.1 caused on average 5.6 ± 5.9 and a visual acuity of ≤0.1 caused on average 11.9 ± 14.2 caring hours per week

On average, the caring time amounted to 6.4 ± 8.5 h per week with a minimum of 0.25 and a maximum of 55 h per week. In a comparison between male and female caregivers, it was shown that female caregivers spend more time on care with 6.86 h per week ± 9.37, compared to the male caregivers with an average of 5.59 h per week ± 6.89. In addition, the relatives reported the caregiving time for various activities in the day-to-day life of the patient. Most of the time was spent on household assistance (4.2 h), leisure activities (1.1 h) and office work (0.5 h) on average per week. The caring hours per week depended on the visual acuity of the patient. In Fig. 1 the significant interdependence between the absolute number of caring hours per week and the visual acuity (Kruskal-Wallis *p*-value <0.01) is shown. A visual acuity of >0.3 caused on average 4.3 ± 4.4, a visual acuity of ≤0.3 - >0.1 caused on average 5.6 ± 5.9 and a visual acuity of ≤0.1 caused on average 11.9 ± 14.2 caring hours per week. This is demonstrated in the boxplots in Fig. 1.

The caregivers were also asked to note the time they spent accompanying the patient to medical treatments. The time spent accompanying the patient to appointments for intravitreal injections took an average of 14.5 h a year. Since 35 % of the caregivers were employed, the leave these caregivers subsequently had to take in order to care for the patient amounted to an average of 3 complete days a year. The specific caring activities and caring times of the caregivers are shown in Table 2.

**Table 2:** Caring activities and caring times

Caring activities and caring times of the caregivers in different time periods	Total sample (*n* = 150)		Visual acuity >0.3 (*n* = 92)		Visual acuity ≤0.3 - >0.1 (*n* = 26)		Visual acuity ≤0.1 (*n* = 32)	
	Mean	SD	Mean	SD	Mean	SD	Mean	SD
Average number of outpatient visits per year (times/year)	9.4	9.7	7.7	8.4	10.3	8.5	13.6	13.0
Average number of accompaniments for intravitreal injections (times/year)	3.3	3.5	3.3	3.8	2.9	2.7	3.7	3.4
Average time spent for each outpatient (hours/visit)	3.7	2.7	3.7	2.0	3.4	2.2	3.6	2.3
Average time spending for accompanying for intravitreal injections; (hours/visit)	3.6	2.1	3.7	2.0	3.4	2.2	3.6	2.3
Average time for household assistance (hours/week)	4.2	12.9	1.4	3.4	9.3	27.0	8.1	11.0
Average time per week for leisure activities (hours/week)	1.2	2.9	0.6	1.7	1.5	3.3	2.4	4.5
Average time per week for office work (hours/week)	0.5	1.2	0.4	0.9	0.4	0.7	1.0	1.9

It becomes clear that, especially with a deterioration of a visual acuity from ≤0.3 - >0.1 to a visual acuity of ≤0.1, the caregivers had on average an increasing care intensity in the different caring activities

**Fig. 2:**
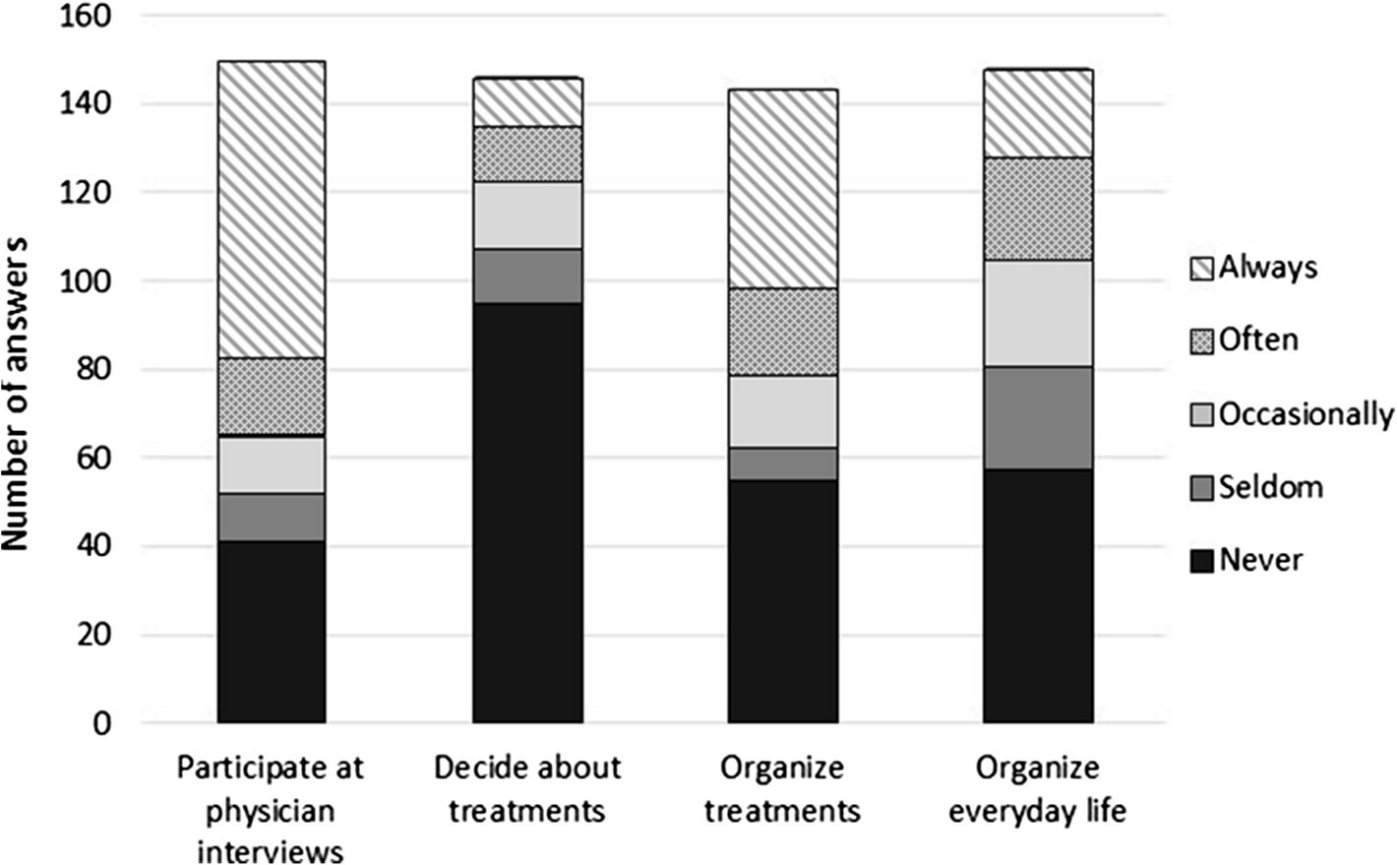
Stacked bar chart with the different mentioned abundances of caring activities. The organization of everyday-life, patient care and participation in discussions with the doctor as well as the decision concerning the patient’s treatment were included in the questionnaire. The questions were answered based on a 5-point Likert scale (1 = never, 2 = seldom, 3 = occasionally, 4 = often, 5 = always). The question about the caregiving concerning the everyday-life of the patient because of the wet AMD was answered mostly with “never”. However, of the total answers marked ‘always’, 88.8 % was a female caregiver and 11.1 % was male. For the answers marked ‘often’, 77.27 % was female and 22.73 % was male. Concerning the everyday-life caregiving of the patient, a significant gender difference could be shown by a chi-squared test (*p* = 0.02). When it comes to the organization of the treatment of wet AMD, 37.3 % indicated that the organization of treatment had ‘seldom’ taken place. However, of the 30.6 % of the total sample that reported that they ‘always’ organized the treatment, 80.4 % were women and 19.5 % were men. The participation of the caregiver at doctors appointments concerning the wet AMD was also requested. The caregivers answered in 45.3 % of the cases that they were ‘always’ involved in the appointments. This ‘always’ was given by 74.6 % of female caregivers and by 25.4 % of male caregivers. Also, the response option ‘often’ was given by 68.7 % of women and by 31.2 % of men. Here a significant gender difference could be shown by a chi-squared test (*p* = 0.03)

The care activities were different with regard to the gender of the caregiver. The results are shown in Fig. 2.

### Financial strain

**Fig. 3:**
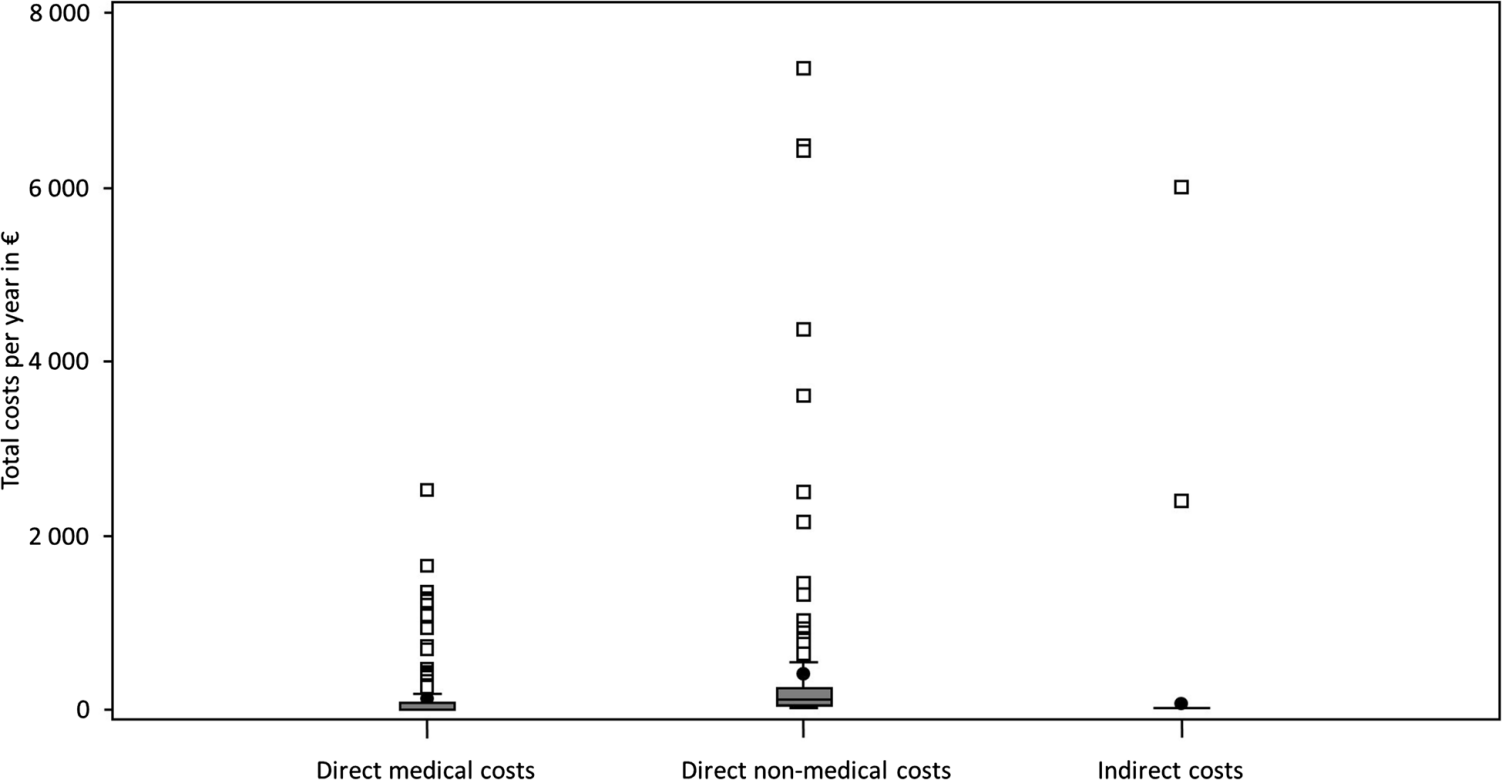
Three different boxplots of the absolute number of the direct medical, direct non-medical and indirect costs of the caregivers. The costs show a skew in the distribution. The most financial strain was brought on by the direct non-medical costs on average € 405 ± € 1103. The direct medical costs amounted to an average of € 134 ± € 340 and two caregivers had indirect costs, namely a loss of net income of € 2400 per year and a loss of € 6000 net income per year. In the total sample, these results had a mean of € 56 ± € 526 per year

The most financial strain was brought on by the direct non-medical costs (travel costs, costs for housekeepers, acquisition costs and modifications). For this purpose, the caregivers spent an average of € 405 ± € 1103 and had a minimum value of € 0 and a maximum of € 7356 per year. The direct medical costs (medical treatment and medication) amounted to an average of € 134 ± € 340 and had a minimum value of € 0 and a maximum value of € 2529 a year. Two caregivers indicated indirect costs due to lost productivity in the workplace. They had to reduce their hours of employment. It was stated by one caregiver that they had a loss of net income of € 2400 per year. The other caregiver stated a loss of € 6000 net income per year. In the total sample, these results have a mean of € 56 ± € 526 per year. Spouses had more total direct costs per year than children (Wilcoxon *p* = 0.013). On average, the costs amounted to € 689 for spouses and € 461 for children. The three different types of costs were very unevenly distributed in the total sample, which can be seen in Fig. 3.

Both the direct medical costs as well as the direct non-medical costs of the caregivers were directly related and both increased significantly by the weekly amount of hours needed to care for the patients. One hour more per week caused € 23 more direct medical costs per year (*R*^2^ = 0.34) and € 62 more non-medical costs (*R*^2^ = 0.23) per year for the relative. However, there was no direct significant relationship between any of the three visual acuities and the rising costs for the caregivers. Nevertheless, as a worse visual acuity caused a higher caring intensity in hours per week, the financial strain was indirectly associated with the visual acuity. A correlation between the caregiver gender and the cost burden or loss of QoL could not be found. Furthermore, to test multivariate effects on the total direct costs, a multivariate ANCOVA was performed with the total direct costs as the dependent variable. Here, the higher caring hours per week, a higher patient age and the fact that the patients were privately insured had a significant influence on the increasing costs, while the caregiver gender, visual acuity of the patient and the caregiver age did not affect the total direct costs shown in Table 3.

**Table 3:** Multivariate ANCOVA of direct total costs, quality of life and caring times

Direct total costs	*p*-value	Caregivers QoL	*p*-value	Caregivers caring time	*p*-value
Multivariate ANCOVA		Multivariate ANCOVA		Multivariate ANCOVA	
Caring hours per week	**<0.001**	Caring hours per week	**<0.001**	Visual acuity	**<0.001**
Health insurance	**<0.01**	Patient age	0.13	Caregivers QoL	**<0.001**
Patient age	**0.02**	Caregiver gender	0.32	Caregiver age	**0.04**
Visual acuity	0.23	Caregiver age	0.54	Patient age	0.40
Caregiver age	0.62	Visual acuity	0.64	Health insurance	0.41
Caregiver gender	0.77	Health insurance	0.79	Caregiver gender	0.80

The costs did increase through patients being privately insured (*p* < 0.01), a higher patient age (*p* = 0.02) and more caring hours per week (*p* < 0.001). Concerning the quality of life of the caregivers, only the caring hours per week had a significant influence (*p* < 0.001). Caregivers caring times were associated with a lower visual acuity (*p* < 0.001), a lower QoL (*p* < 0.001) and a higher caregiver age (*p* = 0.04). Significant data were printed in bold

**Fig. 4:**
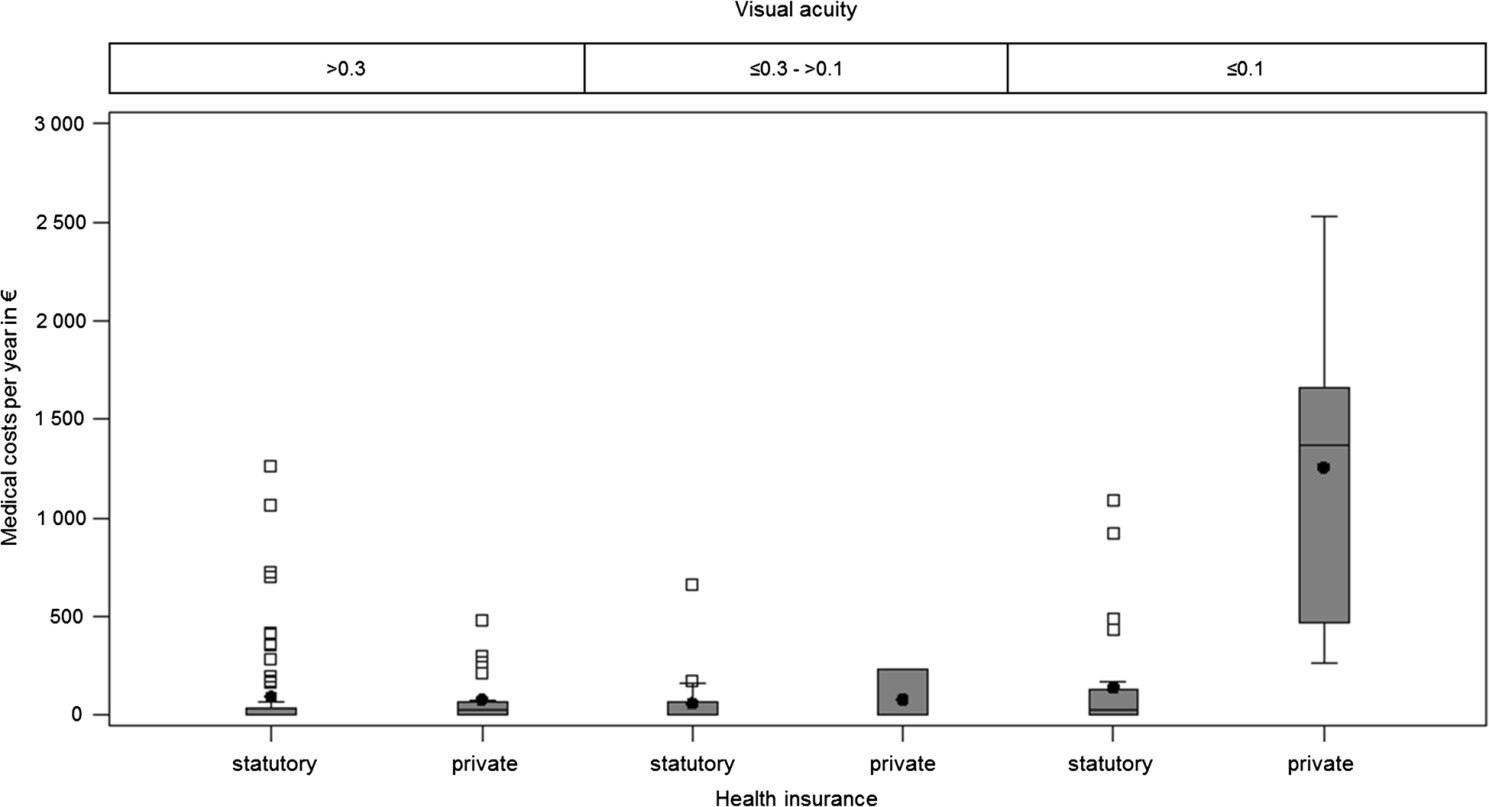
Direct medical costs of caregivers of state (GKV) and privately (PKV) insured patients

**Fig. 5:**
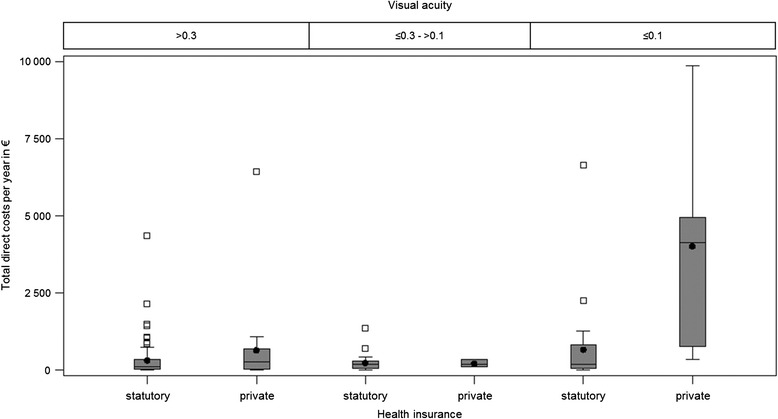
Total direct costs of caregivers of state (GKV) and privately (PKV) insured patients

The analysis showed that the cost burden of the caregivers was associated with whether the patient is privately or state health insured. The caregivers of privately insured patients had significantly higher total direct costs than the others (Wilcoxon *p* = 0.04). On average, the caregivers of state insured patients bore total direct costs of € 385 and caregivers of privately insured patients an average total of € 1207 per year. Also the medical costs of caregivers of privately insured patients were higher (Wilcoxon *p* = 0.03). However, by analysing the different visual acuity of the patients, it was clear that there was no dependence concerning the visual acuity. Caregivers of privately insured patients with the same visual acuity of >0.3 bore double the costs. On average, caregivers of privately insured patients bore costs of € 651 a year, whereas those of state insured patients bore an average of € 324 a year. Caregivers of patients with the same visual acuity of ≤ 0.1 paid an average of € 4024 a year for privately insured patients and an average of € 672 a year for state insured patients, i.e. an almost six times greater cost burden. These increased caregiver costs of privately insured patients could neither be explained by the patients’ visual acuity nor by a higher caregiver income. Also, there was no association with more caring hours per week for caregivers of privately insured patients that could explain the higher costs. Particularly since the Median of the direct medical costs, was much higher for caregivers of privately insured patients. The different caregiver costs are shown in the Appendix 1 in Fig. 4 and Fig. 5. The cost overview is shown in Table 4.

**Table 4:** Total direct and direct medical costs per year in € of caregivers of privately or statutory insured patients

Visual acuity	Health insurance	*N*	Variable	*N*	Mean €	Median €	SD €	Min. €	Max. €
>0.3	Statutory	72	Direct medical costs	72	93	0	236	0	1,266
			Direct total costs	72	324	106	628	0	4,370
	Private	20	Direct medical costs	20	79	28	132	0	480
			Direct total costs	20	651	260	1,407	0	6,444
≤0.3 - >0.1	Statutory	23	Direct medical costs	23	60	3	141	0	666
			Direct total costs	23	242	186	295	0	1,374
	Private	3	Direct medical costs	3	80	6	133	0	233
			Direct total costs	3	217	186	110	126	339
≤0.1	Statutory	27	Direct medical costs	27	140	27	280	0	1,094
			Direct total costs	27	672	202	1,306	10	6,658
	Private	5	Direct medical costs	5	1,258	1,367	921	265	2,529
			Direct total costs	5	4,024	4,152	3,853	358	9,886

Caregivers of privately insured patients had more costs (*p* = 0.04). Despite the same visual acuity of ≤0.1 the caregiver costs of privately insured patients were six times higher, than the caregiver costs of patients with public health insurance, seen in the mean score or 20 times higher seen in the median

By splitting the costs into the three different visual acuities one observes that the third group incurs the highest cost burden for the caregivers. However the increase in burden is not continuous as shown in the Appendix 2 in Table 5.

**Table 5:** Costs in € per year and intangible costs (quality of life) measured by the Visual analog scale (VAS)

Costs in € per year and intangible costs (QoL) measured by the Visual analog scale (VAS)		Total sample (*n* = 150)		Visual acuity >0.3 (*n* = 92)		Visual acuity ≤0.3 - >0.1 (*n* = 26)		Visual auity ≤0.1 (*n* = 32)	
		Mean	SD	Mean	SD	Mean	SD	Mean	SD
Total direct costs (€/year)		539	1,265	395	860	239	278	1,196	2,208
Direct medical costs (€/year)		133	336	90	217	62	138	314	588
Direct non medical costs (€/year)		405	1,104	305	848	176	254	881	1,780
Intangible costs (VAS)		6.7	1.9	6.8	2.0	7.2	1.7	6.2	1.7
QoL of caregivers living in the same household	(*n* = 65)	6.6	2.0						
QoL of caregivers not living in the same household	(*n* = 83)	6.9	1.8						
QoL of sons	(*n* = 19)	6.7	2.2						
QoL of daughters	(*n* = 40)	6.9	1.9						
QoL of female spouses	(*n* = 41)	6.3	1.8						
QoL of male spouses	(*n* = 29)	6.6	1.7						

The values are divided in the total sample and the three different visual acuities. It becomes clear that total direct costs, direct medical and direct non-medical cost increase with a lower visual acuity apart from the visual acuity >0.3 to the visual acuity ≤0.3 - >0.1. Concerning the intangible costs of the different caregivers, it gets clear, that there is no significant strain difference

### Quality of life

The QoL of the caregivers, which has been captured by the VAS, was given on a scale from 0 “totally unhappy” to 10 “perfectly happy”. Here, the mean value was 6.73 ± 1.90. The minimum value was 2 and the maximum value 10. Male caregivers had an average value of 6.93 ± 1.95 and female caregivers an average value of 6.61 ± 1.87.

In a multivariate ANCOVA with the dependent variable of the QoL through the VAS, several variables were tested for their influence on the caregivers’ QoL, which can be seen in Table 3. Here, the caring hours per week were the only variable that had a significant negative impact on the caregivers’ QoL. Increased hours of care per week meant the QoL decreased (*p* < 0.001). The gender of the caregiver, the visual acuity of the patient, the insurance of the patients, the patient age and the caregiver age had no significant effect on the QoL of the caregiver.

## Discussion

Rising costs in the healthcare system mean that a balance needs to be struck between the medical options, their financial sustainability, as well as quality and equity. Consequently, scientific methods in health economics support decision-making in healthcare. The aim is to establish a relationship between the added benefit of an intervention and the scarcity of resources incurred. As wet AMD is mainly treated by very expensive intravitreal injections (VEGF inhibitors), the question is: to what extent are the persons concerned actually burdened? For these reasons, the detection of strain on those affected and their caregivers plays an ever greater role. The study of Bonastre in France [11] described the economic impact of AMD and assessed its medical and non-medical costs out of the perspective of 105 French patients. In this study a significant difference in the direct non-medical costs but not in the direct medical costs between patients with wet AMD with different visual acuity was found. The study of Javitt [16] analysed the association between vision loss and higher medical care costs of patients in the USA. In this context it was found that a higher severity level of visual acuity caused higher medical costs. In addition to the mentioned costs of patients with vision loss, there are also interesting findings concerning costs of informal caregivers. For example the study undertaken by Schmier et al. in the USA [17] investigated the impact of visual impairment on use of caregiving by individuals with AMD, whereby 803 respondents were interviewed. It observed a tendency of increased caregiver time costs simply with increased vision loss, which was also confirmed by our research (*p* = 0.002). The study of Strawbridge in the USA [18] assessd the impact of older spouses’ vision impairment on the health and well-being of their partners and tested gender differences on 418 older couples. It encountered a higher psychological stress level for female caregivers. We were unable to discern a significant difference in the QoL when looking at the caregivers’ gender (*p* = 0.32), even though the female caregivers had a slightly lower QoL on average. Due to the recent literature on caregivers, we could assume that caregivers have high costs and a low Quality of Life when caring for a patient with wet AMD. However, the actual burden of the caregivers in this sample is relatively small and lower than expected. We could verify a rising financial burden and a lower QoL, which was associated with increasing caring hours. However, the total amount of direct medical and non-medical cost of – on average – € 134 and € 405 per year respectively, is lower than in the previous findings. In our study, no further significant differences in QoL or direct medical and non-medical costs among the three visual acuity groups could be proved. It was particularly surprising that the caregiver income had no influence on the caregiver cost burden but rather the insurance of the patients. Even though the group size of publicly and privately insured patients in this study was not the same, it was significant that privately insured patients caused a higher cost burden for caregivers. The fact that the cost burden achieved a twice and almost six times higher amount in the same visual acuity-group was not expected.

In this study a high number of informal caregivers and different new findings have been achieved. The limitation can be seen in the missing comparison group and in the fact, that the caregivers were interviewed only once which makes it difficult to remember the incurred costs of the different time periods. Concerning the fact, if the patient is statutory or privately insured, it is not possible to transfer the findings to other European countries, because of the different reimbursements of costs.

## Conclusions

In conclusion, our study demonstrated that caregivers of patients with wet AMD have a relatively low financial burden compared to the previous findings. Espesially the fact that the financial burden was mainly associated with the privately insured patients and not with the visual acuity, showed a new perspective concerning the health equity context for patients with wet AMD. From the point of view of helath policy in Germany, the results of this study should be taken into account since the higher costs of caregivers of privately insured patients were especially caused by medical aids. It is therefore necessary to question the quality of patient care with medical aids of statutory insured patients. Furthermore the patient care concerning the personal services and rides to the medical treatments musst be covered by the health insurance, since the caring hours per week had a significant negative impact on the caregivers` QoL.

## Appendix 1

ᅟ

## Appendix 2

ᅟ
